# Human Melioidosis, Malawi, 2011

**DOI:** 10.3201/eid1906.120717

**Published:** 2013-06

**Authors:** Thembi Katangwe, Janet Purcell, Naor Bar-Zeev, Brigitte Denis, Jacqui Montgomery, Maaike Alaerts, Robert Simon Heyderman, David A.B. Dance, Neil Kennedy, Nicholas Feasey, Christopher Alan Moxon

**Affiliations:** Queen Elizabeth Central Hospital, Blantyre, Malawi (T. Katangwe, N. Bar-Zeev, N. Kennedy, C.A. Moxon);; University of Malawi College of Medicine, Blantyre (J. Purcell, N. Bar-Zeev, B. Denis, J. Montgomery, M. Alaerts, R.S. Heyderman, N. Feasey, C.A. Moxon);; Liverpool School of Tropical Medicine, Liverpool, UK (R.S. Heyderman, J. Montgomery, C.A. Moxon);; University of Liverpool, Liverpool (N. Bar-Zeev, N. Feasey, C.A.Moxon);; Mahosot Hospital, Vientiane, Lao People’s Democratic Republic (D.A.B. Dance);; University of Oxford, Oxford, UK (D.A.B. Dance);; Wellcome Trust Sanger Institute, Cambridge, UK (N. Feasey)

**Keywords:** Melioidosis, Burkholderia pseudomallei, Africa, osteomyelitis, children, infant, bacteria, Malawi

## Abstract

A case of human melioidosis caused by a novel sequence type of *Burkholderia pseudomallei* occurred in a child in Malawi, southern Africa. A literature review showed that human cases reported from the continent have been increasing.

Melioidosis is widely distributed in tropical and subtropical regions, but data are lacking on this disease in sub-Saharan Africa. We report a case of melioidosis caused by a novel sequence type in a 16-month-old boy from rural Malawi. Increasing reports of melioidosis from Africa indicate a need for further investigation.

## Case Report

A 16-month-old boy was seen at Queen Elizabeth Central Hospital (QECH), Blantyre, Malawi, in March 2011 with fever of 18 days’ duration, poor feeding, subcutaneous lesions of 15 days’ duration, and edema of the hands and feet. He had received intravenous (IV) benzylpenicillin and gentamicin for 4 days at a local health center before being referred for persistent fever.

The family lived in a remote village in the Shire Valley near the Malawi–Mozambique border at a latitude of –15°S; daytime temperature average was 27°–29°C, and natural flooding occurs from October through April. The boy’s parents were subsistence maize farmers and kept goats and pigs.

On arrival at QECH, the child was irritable and pale; temperature was 38.7°C, weight 9.8 kg (weight-for-age z score −1.14), and height 79 cm (weight-for-height z score −1.06). He had bilateral dactylitis with arthritis of the metacarpophalangeal and interphalangeal joints of the lateral 3 fingers. Numerous rubbery, tender subcutaneous nodules of ≈2 cm diameter were palpable on the face, thorax, and limbs. Overlying hyperpigmentation and weepy ulcerations occurred over some nodules. There was cervical and inguinal lymphadenopathy but no hepatosplenomegaly. Symmetric bipedal pitting edema extended to the knees. Neurologic, cardiovascular, and respiratory examinations revealed no abnormalities.

Laboratory results were as follows: blood glucose 7.8 mmol/L (reference 3.5–7.7 mmol/L), hemoglobin 4.6 g/dL (reference 9.7–15.1 g/dL), leukocyte count 31.9 × 10^3^/µL (reference 3.9–10.7 × 10^3^/µL), and erythrocyte sedimentation rate 95 mm/h (reference 3–13 mm/h). Blood smear showed poikilocytes with some tear drops and reticulocytes and was negative for malaria parasites. HIV test (Unigold; Trinity Biotech, Bray, Ireland) and VDRL (Venereal Disease Research Laboratory) test for syphilis were negative. Radiographs of the hand showed bilateral osteolytic reactions in the lateral 3 fingers. Chest radiograph and abdominal ultrasound indicated no abnormalities.

Culture (BacT/Alert PF; bioMérieux, Marcy l’Etoile, France) of blood taken on admission and aspirate of pus from a subcutaneous nodule grew white, oxidase-positive colonies of gram-negative rods, and the biochemical profile (API 20NE; bioMérieux) strongly suggested *Burkholderia pseudomallei* (1556575: *B. pseudomallei* [98.3% identity]). The API profile from the pus isolate (1156154) initially suggested *Chromobacterium violaceum*, a recognized misidentification of *B. pseudomallei*, by API profiling (*1*). Antimicrobial susceptibility by disk diffusion indicated resistance to gentamicin and susceptibility to co-amoxiclav; colistin disk testing was unavailable. Because *B. pseudomallei* has not been reported from Malawi, we sought to confirm the isolate by real-time PCR, targeting the highly specific type III secretion system (*2*). DNA was extracted by using a Wizard Genomic Purification kit (Promega, Madison, WI, USA), and real-time PCR was performed on an Applied Biosystems 7900HT (Applied Biosystems, Foster City, CA, USA) by using a technique modified for SYBR green detection (*2*). This PCR confirmed the identity of the organism as *B. pseudomallei*. Whole-genome sequencing (WGS) was performed by using the MiSeq Personal Sequencer (Illumina, San Diego, CA, USA), which enabled multilocus sequence typing (MLST) (*3*) and revealed a novel allelic combination (1,3,3,1,5,1,1). This sequence type (ST) has been submitted to the MLST database (http://bpseudomallei.mlst.net/) and has been assigned MLST ST1008, part of clonal complex 1.

The boy was given chloramphenicol for empiric treatment of systemic bacterial infection before the isolate was identified. In light of the anthropometric values, anorexia, fecal morphology, and symmetric pedal edema, acute kwashiorkor was diagnosed, and nutritional rehabilitation was begun. Pedal edema and anorexia improved after 48 hours. At 96 hours, *B. pseudomallei* infection was diagnosed, and treatment was changed to IV ceftazidime. Fever abated by day 7, and after 30 days of IV ceftazidime, the nodules had involuted and the dactylitis and arthritis had resolved. At the family’s request, the child was discharged on a 6-month regimen of cotrimoxazole, rather than the planned 6-week IV regimen for osteomyelitis.

Four weeks after discharge, the child remained well with no fevers and no new lesions; clinical anemia had resolved, and repeat radiographs showed that the hands were within normal limits. He was then lost to follow-up.

## Conclusions

Melioidosis is acquired through the skin or possibly by inhalating the environmental organism *B. pseudomallei*. It causes a wide spectrum of clinical disease—from localized skin infection to severe acute septicemia—but progresses to disease in only a small proportion of exposed persons (*4*). *B. pseudomallei* is endemic in Southeast Asia and Northern Australia and typically is distributed from latitude 20°N to latitude 20°S, particularly in association with wet soil (*4*).

Sporadic cases have been documented in all inhabited continents, but a lack of diagnostic microbiological facilities and systematic studies in many low-income regions limit knowledge of the true distribution of the disease (*5*), a particular problem in rural sub-Saharan Africa. Although the relationship between human and animal infection is not precisely understood, infections of animals have been recognized throughout the continent (Table 1) (*6**–**9*). During the past 30 years, 11 cases of human melioidosis acquired in Africa (1 in a child) have been reported in the literature (Table 2 [*10**–**15*; 16–18 in Technical Appendix). Three of these cases were PCR confirmed. In many earlier reports, identification was not confirmed by methods that would satisfy modern taxonomists; thus, the true distribution of melioidosis in Africa remains uncertain.

**Table 1:** Melioidosis in animals, Africa

Year (reference)	Country	Animal	Clinical characteristics	Method of identification
1936 (*6*)	Madagascar	Pig	Lymphadenopathy	Passaged through guinea pig (tissue from submaxillary gland)
1960 (*7*)	Chad	Goat	Lymphadenopathy	Isolated from mesenteric ganglia
1960 (*8*)	Africa*	Camel	Retropharyngeal abscess	Inoculation and sacrifice of guinea pig
1972 (*8*)	Niger, Burkina Faso	Pigs (>100 cases)	Abscesses in liver, spleen, and lung in apparently healthy pigs	Not described
1995 (*9*)	South Africa	Goat	Mammary gland and renal abscesses	Biochemical and phenotypic characteristics

*Specific country of acquisition was not detailed.

**Table 2:** Melioidosis in humans, Africa

Year (reference)	Country where acquired (diagnosed)	Details of infected persons	Clinical characteristics	Diagnostic method (source of isolation)	Definitive treatment (duration, wk)	Outcome
1982 (*10*)	Kenya (Denmark)	Adult	Sepsis	Culture (blood, urine, sputum)	OTC (4); cotrimoxazole TMP-SXT (8)	Complete recovery
1985 (*11*)	Sierra Leone (Gambia)	Child	Cutaneous abscesses, osteomyelitis	Culture; indirect hemagglutination (pus)	CHL, TET	Improved; lost to follow up
2004 (*12*)	Mauritius (Mauritius)	Adult with SLE	Sepsis, cellulitis	Culture; API 2ONE (blood)	None	Died (d 9)
2004 (*13*)	Madagascar (La Réunion)	Adult smoker, alcoholic	Sepsis, respiratory distress	Culture (blood, BAL fluid)	CAZ	Complete recovery
		Adult with CPI	Septicemia	Culture (blood, BAL fluid)	CAZ; cotrimoxazole (20)	Not described
		Adult with SLE	Septicemia	Culture (blood, BAL fluid)	CAZ; cotrimoxazole (20)	Not described
2006 (*14*)	Madagascar (La Réunion)	Adult smoker	Pneumonia	Culture; API 20NE; PCR (BAL fluid);	IPM (2); cotrimoxazole (20)	Resolution at 3 mo
2010 (*15*)	Africa† (France)	Adult	Mycotic aneurysm	Culture (blood, arterial tissue)	IPM + CIP (5); cotrimoxazole (20)	Resolution at 6 mo
2011 (*16*)	Gambia (Spain)	Adult with diabetes mellitus	Pyomyositis, pneumonia	Culture; PCR (pus, sputum)	CAZ + cotrimoxazole (5); DOX + cotrimoxazole (20)	Complete recovery
2011 (*17*)	Nigeria (UK)	Adult with diabetes mellitus	Localized lymphadenopathy	Culture; chromatography; PCR (blood)	Meropenem (1); cotrimoxazole (12)	Lost to follow-up
2011 (*18*)	Africa† (Spain)	Adult	Sepsis	Culture (blood)	CAZ + DOX	Not described

*OTC, oxytetracycline; TMP-SXT, trimethoprim–sulfamethoxazole; CHL, chloramphenicol; TET, tetracycline; SLE, systemic lupus erythematosus; BAL, bronchoalveolar lavage; CAZ, ceftazidime; CPI, chronic pulmonary insufficiency; IPM, imipenem; CIP, ciprofloxacin; DOX, doxycycline; MER, meropenem. †Exposure was in multiple countries on the continent or the specific country of acquisition was not detailed.

We used eBURST software (19 in online Technical Appendix) to model the relationship between ST1008 and the global MLST database (Technical Appendix Figure 1). The founder of this branch of clonal complex 1 is ST916 (Technical Appendix Figure 2), which was isolated from Cambodia. The other STs on this branch, ST186 and ST250, were isolated in Thailand. Although none of the other Africa *B. pseudomallei* isolates of known MLST are predicted to be in the same subgroup, 2 isolates from human infections that are thought to have occurred in Kenya (ST5 and ST9; Technical Appendix Figure 2) are in the adjacent subgroup. Thus, these Malawi and Kenya strains might share a recent common ancestor. We have submitted WGS data from our sample to a project that is undertaking WGS on a large number of *B. pseudomallei* isolates from around the world. This approach is anticipated to offer superior resolution of the global phylogeny.

Unfamiliarity with the culture characteristics of *B. pseudomallei* often has resulted in delays in recognition, identification, diagnosis, and treatment (*1*). The organism exhibits considerable interstrain and media-dependent variability in colonial morphology (*1*); its wrinkled appearance in older colonies may result in their dismissal as contaminants. Even relatively expensive biochemical test kits, such as the API20NE, may result in misidentification, as with the pus isolate here, which raises the question about whether the infrequency of the diagnosis is due to rarity of the disease or lack of capacity to identify it. *B. pseudomallei* might be more widespread than recognized in Malawi and ecologically similar areas of sub-Saharan Africa where the environment is conducive to its growth. Health care in Malawi, as in most of sub-Saharan Africa, is delivered frequently without use of even basic diagnostic facilities, which leads to overdiagnosis of malaria and tuberculosis (20 in Technical Appendix). In this environment, patients with septicemic melioidosis could have died before all locally available empiric treatments had been tried. *B. pseudomallei* is resistant to penicillin, gentamicin, and many other antimicrobial drugs used to treat sepsis in the tropics, so diagnosis is necessary for appropriate antimicrobial therapy.

Melioidosis therefore could be underestimated in Malawi and throughout the region. Environmental microbiology and seroprevalence studies are required to gauge the extent of this infection and to guide local and regional health care policy.

Technical Appendix Additional references and eBURST diagram of clonal complex 1 *Burkholderia pseudomallei* sequence types.

## References

[1] Inglis TJ, Merritt A, Chidlow G, Aravena-Roman M, Harnett G. Comparison of diagnostic laboratory methods for identification of *Burkholderia pseudomallei.* J Clin Microbiol. 2005;43:2201–6. 10.1128/JCM.43.5.2201-2206.2005 15872242 PMC1153801

[2] Novak RT, Glass MB, Gee JE, Gal D, Mayo MJ, Currie BJ, Development and evaluation of a real-time PCR assay targeting the type III secretion system of *Burkholderia pseudomallei.* J Clin Microbiol. 2006;44:85–90. 10.1128/JCM.44.1.85-90.2006 16390953 PMC1351940

[3] Croucher NJ, Harris SR, Fraser C, Quail MA, Burton J, van der Linden M, Rapid pneumococcal evolution in response to clinical interventions. Science. 2011;331:430–4. 10.1126/science.1198545 21273480 PMC3648787

[4] Cheng AC, Currie BJ. Melioidosis: epidemiology, pathophysiology, and management. Clin Microbiol Rev. 2005;18:383–416. 10.1128/CMR.18.2.383-416.2005 15831829 PMC1082802

[5] Dance DA. Melioidosis: the tip of the iceberg? Clin Microbiol Rev. 1991;4:52–60 . 2004347 10.1128/cmr.4.1.52 PMC358178

[6] Girard G. Pigs can be a healthy carrier of Whitmore’s bacillus [in French]. Bull Soc Pathol Exot. 1936;29:712–6.

[7] Provost A, Vigier M. Isolation in Tchad (Central Africa) of 2 strains of *Malleomyces pseudomallei* [in French]. Ann Inst Pasteur (Paris). 1960;98:461–3 . 14435122

[8] Dodin A, Ferry R. Epidemiological studies of the bacillus of Whitmore in Africa [in French]. Bull Soc Pathol Exot. 1974;67:121–6 . 4480518

[9] Van der Lugt JJ, Henton MM. Melioidosis in a goat. J S Afr Vet Assoc. 1995;66:71–3 . 8544164

[10] Bremmelgaard A, Bygbjerg I, Hoiby N. Microbiological and immunological studies in a case of human melioidosis diagnosed in Denmark. Scand J Infect Dis. 1982;14:271–5 . 7163779 10.3109/inf.1982.14.issue-4.05

[11] Wall RA, Mabey DC, Corrah PT, Peters L. A case of melioidosis in West Africa. J Infect Dis. 1985;152:424–5. 10.1093/infdis/152.2.424a 4031552

[12] Issack MI, Bundhun CD, Gokhool H. Melioidosis in Mauritius. Emerg Infect Dis. 2005;11:139–40. 10.3201/eid1101.040605 15705340 PMC3294329

[13] Martinet O, Pac Soo AM, Knezynski M. Melioidosis: Regarding a case acquired in Madagascar and two nosocomial cases [in French]. Bull Soc Pathol Exot. 2004;97:369.

[14] Borgherini G, Poubeau P, Paganin F, Picot S, Michault A, Thibault F, Melioidosis: an imported case from Madagascar. J Travel Med. 2006;13:318–20. 10.1111/j.1708-8305.2006.00050.x 16987131

[15] Amezyane T, Lecoules S, Algayres JP. Mycotic iliac aneurysm associated with *Burkholderia pseudomallei.* Int J Infect Dis. 2010;14(Suppl 3):e381–2. 10.1016/j.ijid.2009.07.008 19897393

