# Supplementary material for: Human Melioidosis, Malawi, 2011
Source: Emerg Infect Dis. 2013 Jun;19(6):981–4. doi: 10.3201/eid1906.120717 (PMC3713813; doi:10.3201/eid1906.120717)
Supplement: Technical Appendix — Additional references and eBURST diagram of clonal complex 1 Burkholderia pseudomallei sequence types. [file 12-0717-Techapp-s1.pdf]

Article DOI: http://dx.doi.org/10.3201/eid1906.120717

Human Melioidosis, Malawi, 2011

Technical Appendix

References 16–20

16. Cuadros J, Gil H, Miguel JD, Marabe G, Gomez-Herruz TA, Lobo B, et al. Melioidosis imported from West Africa to Europe. Clin Microbiol Rev. 2011;85:282–4. PubMed

17. Salam AP, Khan N, Malnick H, Kenna DT, Dance DA, Klein JL. Melioidosis acquired by traveler to Nigeria. Emerg Infect Dis. 2011;17:1296–8. PubMed http://dx.doi.org/10.3201/eid1707.110502

18. Lopez-Velez R. Melioidosis—Spain: (Madrid) ex Africa. ProMEDmail. 2011 Apr 13 [cited 2012 Mar 15]. http://www.promedmail.org, archive no. 20110413.1164.

19. Feil EJ, Li BC, Aanensen DM, Hanage WP, Spratt BG. eBURST: inferring patterns of evolutionary descent among clusters of related bacterial genotypes from multilocus sequence typing data. J Bacteriol. 2004;186:1518–30 PubMed http://dx.doi.org/10.1128/JB.186.5.1518-1530.2004

20. Chandler CI, Mwangi R, Mbakilwa H, Olomi R, Whitty CJ, Reyburn H. Malaria overdiagnosis: is patient pressure the problem? Health Policy Plan. 2008;23:170–8. PubMed http://dx.doi.org/10.1093/heapol/czm046

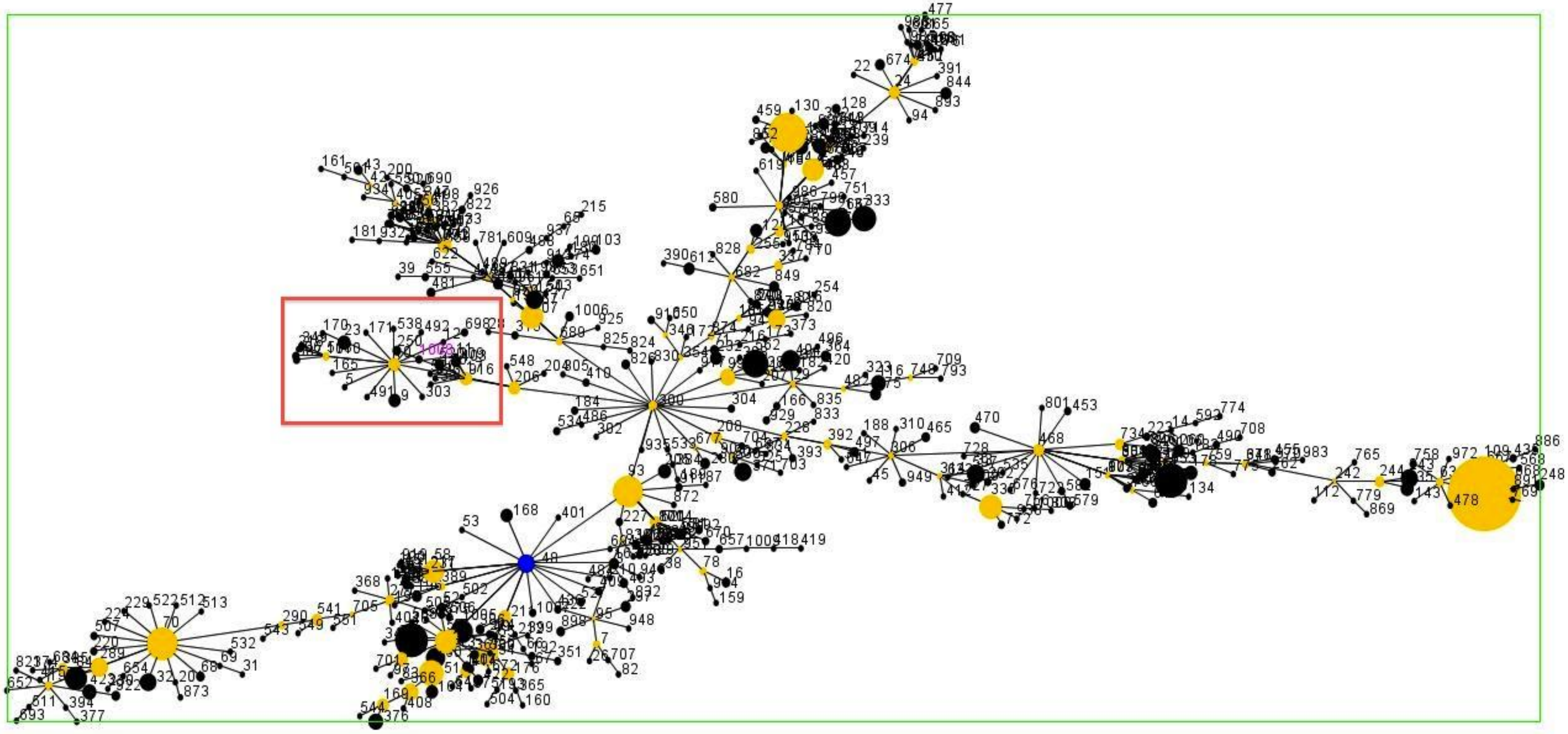

Technical Appendix Figure 1. eBURST diagram of clonal complex 1 *Burkholderia pseudomallei* sequence types (STs) submitted to the global multilocus sequence typing (MLST) database. Diagram shows predicted evolutionary relationship between strains by ST in 7 different alleles. Each circle indicates a particular ST, with the relative size of the circle indicating the number of isolations of that particular ST. Blue circle indicates the predicted founding genotype (ST48); yellow circles indicate predicted subgroup founders; and purple circle indicates ST1008 from the Malawi case described in this report. Lines between STs indicate STs that differ at a single allele. The red square is magnified in Technical

Appendix Figure 2 to illustrate more clearly STs closely related to ST1008. The complete database and the eBURST software can be accessed at http://bpseudomallei.mlst.net/

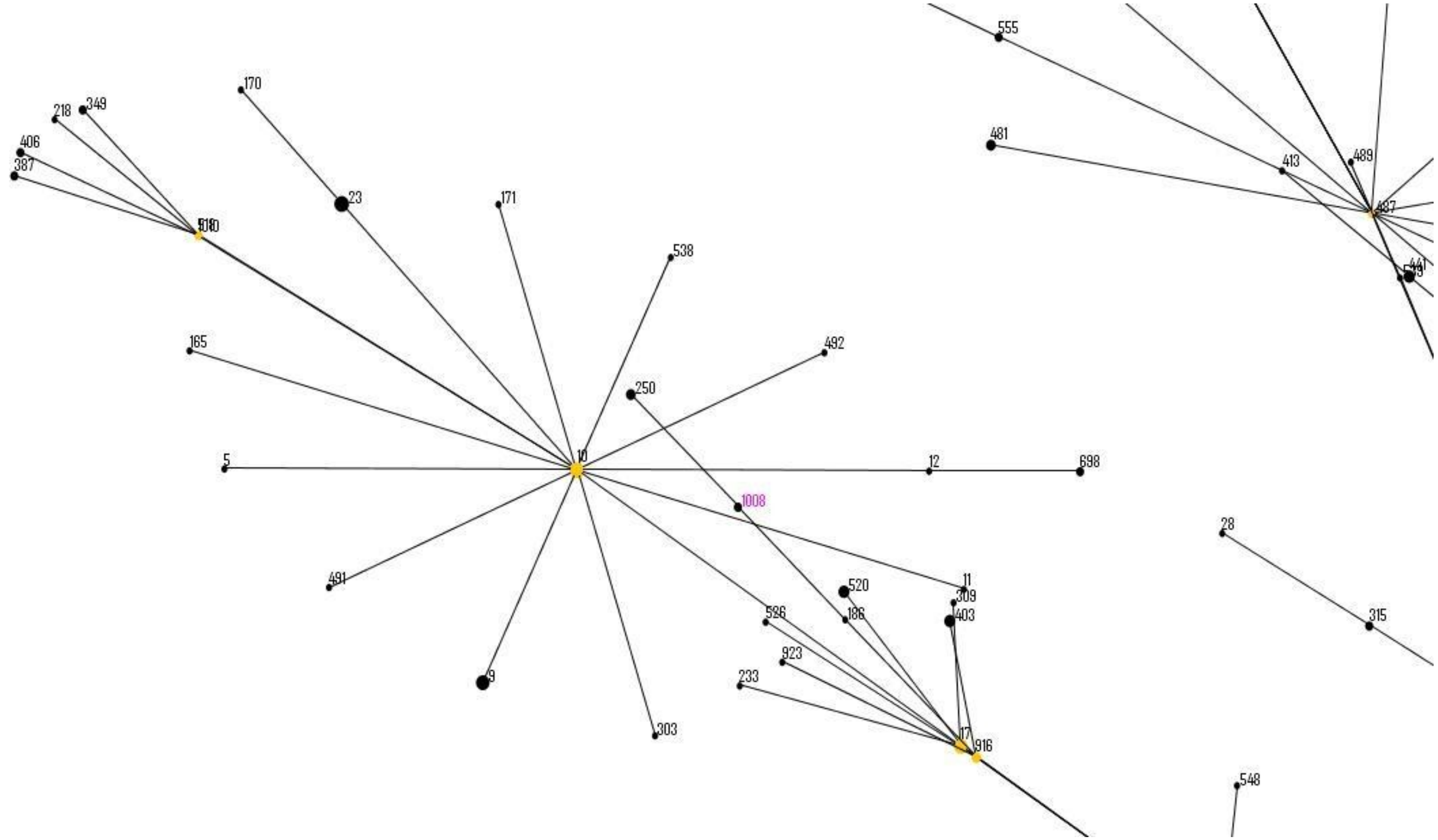

Technical Appendix Figure 2. Detail of eBURST clonal complex 1 subgroup for ST1008. ST1008 is labeled in magenta. The subgroup founder ST916 (bottom right) was isolated from samples in Cambodia. In the adjacent subgroup are STs (ST5 and ST-9) isolated from infections in humans in Kenya.
